# Metal Ion Toxins and Brain Aquaporin-4 Expression: An Overview

**DOI:** 10.3389/fnins.2016.00233

**Published:** 2016-06-01

**Authors:** Adriana Ximenes-da-Silva

**Affiliations:** Setor de Fisiologia, Instituto de Ciências Biológicas e da Saúde, Universidade Federal de AlagoasMaceió, Brazil

**Keywords:** aquaporin-4, neurotoxicity, astrocytes, metal ions, oxidative stress, brain edema

## Abstract

Metal ions such as iron, zinc, and manganese are essential to metabolic functions, protein synthesis, neurotransmission, and antioxidant neuroprotective mechanisms. Conversely, non-essential metals such as mercury and lead are sources of human intoxication due to occupational activities or environmental contamination. Essential or non-essential metal accumulation in the central nervous system (CNS) results in changes in blood-brain barrier (BBB) permeability, as well as triggering microglia activation and astrocyte reactivity and changing water transport through the cells, which could result in brain swelling. Aquaporin-4 is the main water channel in the CNS, is expressed in astrocyte foot processes in brain capillaries and along the circumventricular epithelium in the ventricles, and has important physiological functions in maintaining brain osmotic homeostasis and supporting brain excitability through regulation of the extracellular space. Some evidence has pointed to a role of AQP4 during metal intoxication in the brain, where it may act in a dual form as a neuroprotector or a mediator of the development of oxidative stress in neurons and astrocytes, resulting in brain swelling and neuronal damage. This mini-review presents the way some metal ions affect changes in AQP4 expression in the CNS and discuss the ways in which water transport in brain cells can be involved in brain damage.

## Introduction

Several metals, including zinc, iron, and manganese are important as major or trace elements in cellular biological functions, acting as catalytic cofactors of enzymes (Kress et al., 2002), cellular antioxidants, and neuromodulators (Paoletti et al., 1997). In contrast, metals such as mercury, lead, cadmium, and nickel have no known biological functions. Both essential and non-essential metal ions may lead to brain damage when they accumulate in the central nervous system (CNS).

Non-essential metals are an important cause of human intoxication due to occupational exposure or air, soil, and water contamination, resulting in serious health problems (Valko et al., 2005; Park and Zheng, 2012), including severe hematopoietic, renal, and neurological conditions (Tchounwou et al., 2012). More recently, the role of glial cells in protecting neuronal damage caused by metal ion accumulation in the brain has been studied, showing that astrocytes have a central role in reducing neural excitotoxicity by taking up metals that cross the blood-brain barrier (BBB) (Ni et al., 2011; Noguchi et al., 2013), while microglia release mediators of inflammatory and immune responses when activated by metal ions. As a consequence, oxidative stress is generated in brain cells, and reactive nitrogen and oxygen species (RNOS) contribute to the apoptotic process, leading to neurodegenerative diseases (Yuste et al., 2015).

Metal intoxication often leads to increased water transport through the BBB and astrocytes, which could have important consequences on the expression of aquaporins in the brain. Aquaporins are integral membrane proteins that mediate the bi-directional transport of water through the cells, regulating the osmolarity of the intra- and extracellular medium. Aquaporins possess six membrane-spanning domains and five connecting loops. To date, thirteen main isoforms of aquaporins have been described (AQP0 to AQP12). The isoforms AQP3, 7, 9, and 10 are known as aquaglyceroproteins, which mediate the transport of glycerol, urea, and carbon dioxide in addition to water.

In the CNS, AQP1 is mainly found in the apical membrane of the epithelium of the choroid plexus and in the ependyma and pia (Nielsen et al., 1993), while AQP4 is the main water channel expressed in glial cells (Jung et al., 1994). In astrocytes, AQP4 is localized in the foot processes apposed to brain capillaries and along the circumventricular epithelium in the ventricles (Nielsen et al., 1997). The distribution of AQP4 in astrocyte processes is polarized, and the channels are assembled as orthogonal arrays of particles (OAPs; Yang et al., 1996), which share the same distribution as the inwardly rectifying K^+^ channel Kir4.1 (Nagelhus et al., 1999), showing an expressive role for AQP4 in regulating homeostasis of brain osmolarity and excitability via the extracellular clearance of K^+^.

Therefore, oxidative stress due to metal intoxication seems to have an important role in brain excitotoxicity and damage, with cells swelling as a consequence or cause of neuronal damage. This mini-review aims to bring an overview of the role of AQP4 during metal intoxication and cellular mechanisms involved in neuroprotection and toxicity of the brain.

## Exposure to non-essential metals and the role of astrocyte water transport in brain injury (Table 1, Figure 1)

### Mercury

Human intoxication due to mercury exposure is associated with occupational activities, including mining and smelting of cinnabar ore, environmental pollution, and consumption of seafood contaminated with mercury. Central nervous system symptoms related to mercury exposure include paresthesia, cerebellar ataxia, and decrease of cognition (Ye et al., 2016).

**Table 1:** **Effects of metal-ions on aquaporin-4 (AQP4) expression in the brain**.

**Metal Ion**	**Experimental approach**	**AQP4 expression**	**Water permeability**	**References**
Mercury	*in vivo*			
	Rat brain	↑mRNA ↑protein	–	Yukutake et al., 2008
	*in vitro*			
	*Xenopus* oocytes	–	↓	Shi and Verkman, 1996
	AQP4 cystein-residue mutants			Yukutake et al., 2008
	AQP4 reconstituted in proteoliposomes	–	↓	
Lead	*in vivo*			
	Rat brain	no changes mRNA	–	Gunnarson et al., 2005
	*in vitro*			
	Cultured astrocytes	–	↑	Gunnarson et al., 2005
Manganese	*in vitro*			
	cultured astrocytes	↑protein in plasma membrane	↑	Rao et al., 2010
		no changes mRNA		
Zinc	*in vitro*			
	AQP4.M23 proteolipossomes	–	↓	Yukutake et al., 2009
Iron	*in vivo*			
	Rat brain	↑protein	↑	Qing et al., 2009
	*in vitro*			
	cultured astrocytes	↑protein	–	Wang et al., 2015

↑, Increased; ↓, Decreased.

**Figure 1:**
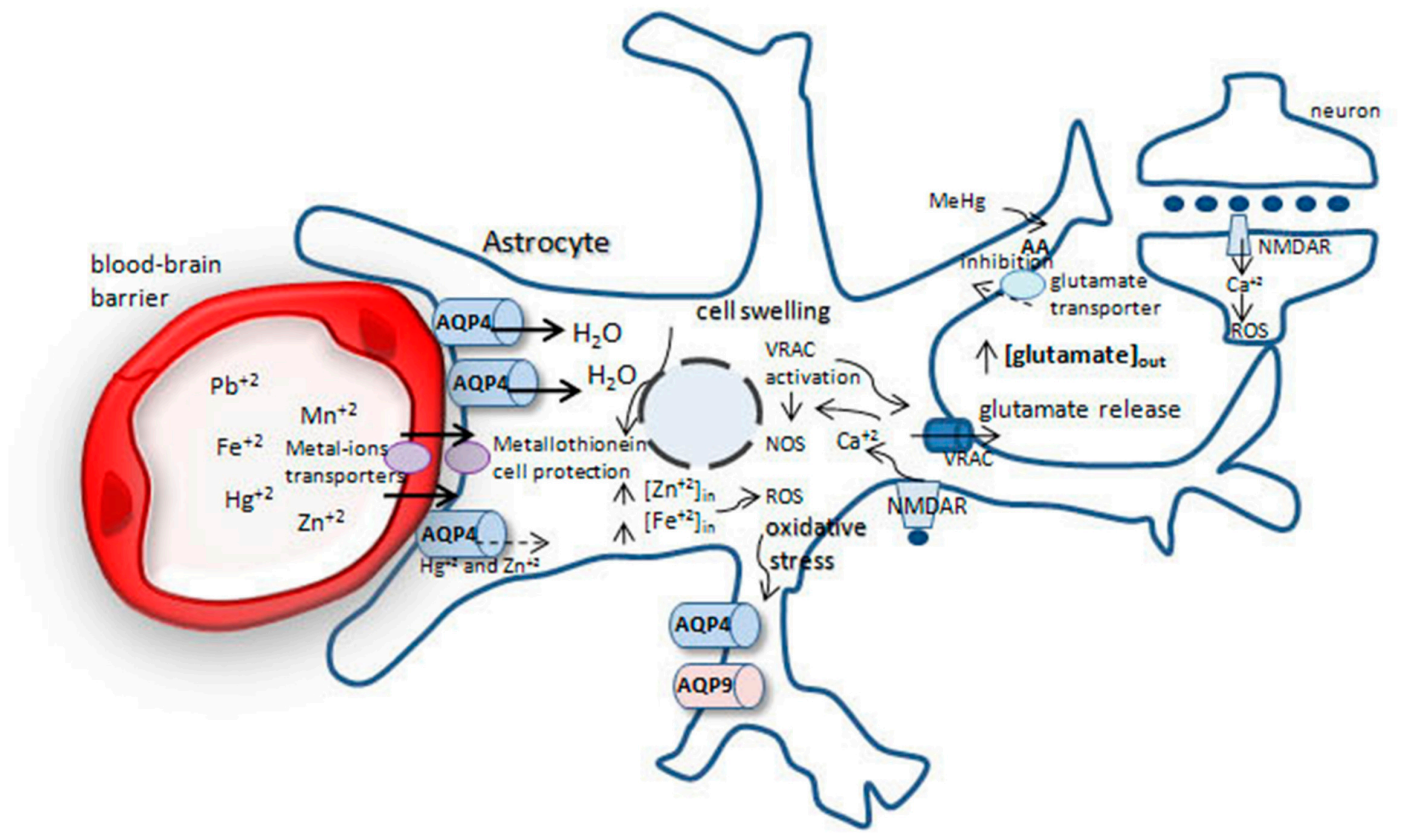
**Possible mechanisms involved in brain protection or swelling after metal-ion intoxication**. After crossing the blood-brain barrier metal-ions are taken up by astrocytes. Fe^+2^, Mn^+2^, Pb^+2^ increase water permeability throughout aquaporin-4 (AQP4), leading to cell swelling and glutamate release from astrocytes by volume-regulated anion channels (VRACs), increasing extracellular glutamate pool. Glutamate acts via NMDA receptor in neurons and astrocytes, increasing [${\text{Ca}}_{\text{i}}^{+2}$] and reactive oxygen species (ROS) production, generating oxidative stress. Methylmercury (MeHg) reduces cystein uptake, increasing ROS production. Oxidative stress leads to increase in AQP4 and AQP9 in astrocytes, which leads to cell swelling.

Elemental mercury is lipid-soluble and crosses the BBB by diffusion. In brain cells, it is oxidized through catalase and peroxidase to inorganic mercury (Hg^+^ and Hg^2+^). Methylmercury (MeHg) is an important source of human intoxication through seafood intake. MeHg is carried through the endothelial cells of the BBB by a neutral l-cysteine amino acid carrier, and is preferentially taken up by astrocytes and microglia. In astrocytes, MeHg promotes RNOS production through decreased availability of cysteine, reducing the antioxidant responses. Moreover, MeHg stimulates arachidonic acid synthesis, which in turn inhibits glutamate uptake by astrocytes, leading to neurotoxicity through ROS production (Ni et al., 2012).

Aquaporins are mercury-sensitive water channels. Most AQPs are inhibited by Hg^2+^, therefore decreasing cell water permeability (Agre et al., 1998), except for AQP6, which is activated by Hg^2+^ exposure (Yasui et al., 1999). The effects of mercury on cell's water transport were described in an early study in ghost erythrocytes treated with p-chloromercuribenzene sulphonate. In erythrocytes, mercury inhibited ~36% of water diffusion (Benga et al., 1985).

AQP4 has two isoforms (M1 and M23) that have been described as mercurial-insensitive water channels (MIWC), since their osmotic water permeability was not inhibited by mercury compounds (Shi and Verkman, 1996). In cells, the AQP4 monomer is normally oriented with the cysteine residue in loop D facing the intracellular side, preventing mercury binding to the channel and rendering AQP4 mercury-insensitive. However, mutagenic assays directed to the cysteine residues in the rat AQP4M23 isoform in proteoliposomes, in which the cysteine residue in loop D was randomly positioned inside or outside the proteoliposome membrane, demonstrated that when the AQP4M23 Cys178 in loop D faced the outside of proteoliposomes, mercury was efficient in reducing water permeability through AQP4. Covalent binding of mercury to the Cys178 residue might induce conformational changes in the AQP4 monomer, reducing water permeability (Yukutake et al., 2008). One *in vivo* study demonstrated the effects of 14 days of MeHg exposure (1.5 mg Hg/kg/day p.o.) on AQP4 expression in the brain of marmosets. MeHg intoxication increased AQP4 mRNA in the frontal lobe, occipital lobe, and cerebellum, while the AQP4 protein was increased in the occipital lobe and cerebellum (Yamamoto et al., 2012).

### Lead

Lead (Pb) intoxication effects in the CNS include lethargy, memory deficits, encephalopathy, and coma. Lead poisoning in humans occurs by breathing dust or swallowing paint, water, or food containing lead. When bound to sulfhydryl groups of hemoglobin, lead reaches brain vessels and crosses the BBB by diffusion or competition with the same carrier system for other metal ions, as iron. Mechanisms of lead intoxication are mainly related to ROS production and disturbed ionic mechanisms, as lead can substitute for bivalent cations as Ca^+2^, Mg^+2^, and Fe^+2^, leading to neurotransmission deficits, impaired subcellular signaling, and oxidative stress (for review, see Needleman, 2004).

Lead intoxication changes BBB permeability as a result of its accumulation in endothelial cells, leading to capillary weakness and brain swelling. Cultured rat astrocytes exposed to lead showed a 40% increase in osmotic water permeability (*P*f) in AQP4-expressing astrocytes. The lead chelator DMSA (meso-2, 3-dimercaptosuccinic acid) abolished the effects of lead on water permeability, showing that AQP4 in astrocytes is central to cell swelling after lead intoxication (Gunnarson et al., 2005).

Changes in astrocyte water permeability would be caused by the calcium/calmodulin-dependent protein kinase II (CaMKII) pathway; astrocyte exposure to a CaMKII inhibitor abolished the lead effects on water permeability. The AQP4 phosphorylation site for CaMKII is located at the Ser111 residue; when mutated to Ser111Ala, the effect of lead on water permeability was prevented. Assentoft et al. (2013, 2014) have questioned the effect of Ser111 residue phosphorylation on AQP4's regulation of water permeability. Mutation of the Ser111Ala residue to abolish the potential site of AQP4 phosphorylation and mutation to aspartate (S111D) to mimic serine phosphorylation did not change water permeability in *Xenopus* oocytes. Similarly, primary culture of astrocytes exposed to a cGMP-dependent protein kinase (PKG) activator did not change water permeability, indicating that phosphorylation of AQP4 could not be implicated in cell swelling. Another *in vivo* study in Sprague-Dawley rats did not indicate lead-related changes in AQP4 expression. Ten and fourty day old rats that received lead acetate intraperitoneally or by gavage showed no difference in AQP4 mRNA in the cerebellum and cerebrum at either age, although significantly increased brain lead levels could be detected (Gunnarson et al., 2005).

## Biologically-necessary metals and the role of astrocyte water transport in brain injury (Table 1, Figure 1)

### Manganese

Manganese is an essential metal and a constituent of metalloproteins and mitochondrial enzymes in oxidative metabolism (Aschner, 2000). Manganese poisoning occurs mainly through occupational exposure of miners, industrial steel workers, or welders to heavy metals. High exposure to manganese results in neurological symptoms, including bradykinesia, dystonia, and gait disturbance. At a cellular level, manganese poisoning will disturb antioxidant defense and water transport in cells, leading to swelling (Erikson et al., 2004). Primary cultures of rat astrocytes treated with manganese showed increased AQP4 proteins in the plasma membrane. This effect was time-dependent, and there was no corresponding increase in mRNA. Conversely, astrocyte cultures transfected with siRNA targeted to AQP4 showed a significant reduction (~86%) of astrocyte swelling mediated by the AQP4 protein when exposed to manganese. The effects of manganese on cell swelling seem to involve mitogen-activated protein kinases (MAPKs) in astrocytes, since inhibition of ERK1/2/3 and p38-MAPK prevented AQP4 protein increases in the plasma membrane (Rao et al., 2010).

### Zinc

Zinc is an essential trace element for all cells, involved in various metabolic and signaling pathways as component of regulatory and catalytic proteins (Mizuno and Kawahara, 2013). In the brain, zinc is mostly bound to proteins and has important modulatory functions in glutamatergic synapses (Tamano and Takeda, 2011). Zinc intoxication is a consequence of inhalation, ingestion, or manipulation of metal. Free intracellular Zn^2+^, which is present during intoxication, generates oxidative stress in neurons and astrocytes and modulates neuronal activity.

In the brain, Zn^2+^ is mainly distributed in membrane-bound metalloproteins and presynaptic vesicles in glutamatergic neurons. When the amount of free Zn^2+^ increases in the brain, oxidative stress is triggered and activates nitric oxide synthetase (NOS), which in turn releases Zn^2+^ from intracellular stores and activates apoptosis. Increases in free Zn^2+^ promote cellular swelling (Kruczek et al., 2009). Cultured rat astrocytes exposed to a hypo-osmotic milieu (205 mosm/L) increased Zn^2+^ concentrations in the cytoplasm, mitochondria, and nucleus. Hypo-osmotic-dependent zinc increase in astrocytes seems to be trigged by Ca^2+^ and ROS intracellular signaling, as antagonists of the NMDA receptor prevent hypo-osmotic Zn^2+^ increase (Kruczek et al., 2009). Hypo-osmotic effects in astrocytes can be mediated in part by the recently identified AQP4e isoform (Moe et al., 2008). Rat astrocytes transfected with AQP4e and maintained in hypo-osmolar solution (200 mosm/L) showed a transitory increase of AQP4e membrane insertion, concomitant with diminished mobility of the AQPe-carrying vesicles. Depolymerization of vimentin filaments in the cytoskeleton under hypo-osmotic conditions would contribute to the AQPe mobility and membrane insertion (Potokar et al., 2013).

Zinc seems to have an inhibitory effect on water permeability, as demonstrated in AQP4.M23 expressed in proteoliposomes. The Cys178 residue in AQP4 is a potential site for the inhibitory effect of zinc on water permeability, since mutation of this residue resulted in no change in water permeability after zinc exposure (Yukutake et al., 2009).

### Iron

Iron is an essential metal in multiple metabolic reactions, including DNA synthesis, enzymatic reactions, and electron transport. Iron accumulation is very deleterious for brain functions due to its wide participation in metabolic reactions (Schipper, 2012; Rouault, 2013). Iron intoxication is not common; however, iron deposition in cells occurs frequently after intracerebral hemorrhage (ICH), a subtype of stroke with high morbidity and mortality in humans.

As early as 24 h after ICH, iron content increases in the perihematomal zone and peaks at day 7. AQP4 expression peaks at day 3 and is maintained until day 7. Brain water content follows the initial increase of AQP4 and then declines slowly until day 14 post ICH onset. AQP4 expression is increased in astrocytes near the perihematomal area. The iron chelator deferoxamine (DFO) reduced iron deposition, brain water content, and AQP4 level in the perihematomal area, demonstrating a correlation between free iron content and brain swelling mediated by AQP4. Additionally, increased iron deposition and brain water permeability are likely to initiate apoptosis in perihematomal areas (Qing et al., 2009).

Cell damage as a result of increased Fe^2+^ is mediated by the NF-κB p65 protein, which activates ROS production and release of proinflammatory cytokines in astrocytes and microglia, respectively, and consequently increases AQP4 and AQP9 in astrocytes (Wang et al., 2015). These studies evidenced increased iron-dependent water permeability in astrocytes mediated by AQP4, showing a role for free iron brain deposition and increased risk of brain damage.

## Conclusions

Metal ions including iron, zinc, and manganese are essential to metabolic functions, protein synthesis, neurotransmission, and antioxidant neuroprotective mechanisms. However, in the CNS, unbalanced essential metal ion amounts, as well as non-essential metal accumulations, are detrimental to brain function.

Toxic amounts of non-essential metals and breakdown of metal ion homeostasis result in changes in brain metabolism and water permeability. These changes are particularly related to increased AQP4 expression in the astrocytes surrounding the BBB, the development of oxidative stress in neurons and astrocytes, and brain swelling, leading to neurodegeneration.

## Author contributions

The author confirms being the sole contributor of this work and approved it for publication.

### Conflict of interest statement

Conflict of Interest Statement: The author declares that the research was conducted in the absence of any commercial or financial relationships that could be construed as a potential conflict of interest.
